# Mapping TAM–tumor crosstalk in glioma via ligand–receptor multi-omics: mechanisms of immune evasion

**DOI:** 10.3389/fimmu.2025.1699915

**Published:** 2025-11-21

**Authors:** Dong Zhang, Yiming Ma, Daxiong Feng

**Affiliations:** 1 Department of Orthopedics, The Affiliated Hospital of Southwest Medical University, Luzhou, China; 2 Department of Orthopedics, The Second People’s Hospital of Neijiang, Neijiang, China; 3 Department of Neurology, The Second People’s Hospital of Neijiang, Neijiang, China

**Keywords:** glioma, tumor-associated macrophages, microglia, ligand–receptor signaling, single-cell RNA sequencing, spatial transcriptomics, immune evasion

## Abstract

Diffuse gliomas remain lethal primary brain tumors. Immune-checkpoint inhibitors have not delivered durable benefit for most patients, reflecting myeloid-dominant immunosuppression and spatially organized immune exclusion. In this mini-review we summarize ligand–receptor multi-omics—single-cell RNA/CITE-seq, single-cell chromatin accessibility, and spatial proteo-transcriptomics—that resolve microglia- and monocyte-derived TAM programs and malignant state continua, and we appraise translational opportunities spanning TAM reprogramming (CSF1–CSF1R), perivascular SPP1–CD44 disruption, and innate–adaptive combinations targeting CD47–SIRPα, CD39–CD73, and PD-1/PD-L1. We also discuss challenges—including ontogeny-aware state definitions, heteromer-aware databases, chromatin gating of receivers (requiring accessible regulatory DNA for the receptor and its program), spatial registration, and limited assay standardization—that temper implementation. By integrating myeloid-informed readouts (SPP1–TAM burden, CD39–CD73 proximity, HMOX1+ IL-10 niches, serum IL-8), emerging strategies aim to restore antigen presentation, enable effector ingress, and remodel vascular–stromal interfaces. Our synthesis provides an appraisal of reproducible communication architectures in glioma and outlines pragmatic reporting standards and trial-ready pharmacodynamic endpoints for myeloid-informed precision immuno-oncology. We hope these insights will assist researchers and clinicians as they design multi-omics pipelines and interventions to convert suppressive ecosystems into responsive ones.

## 1 Introduction

Diffuse gliomas develop in an immune microenvironment dominated by tumor-associated macrophages (TAMs) from brain-resident microglia and infiltrating monocytes; these lineages regulate tumor growth, therapy resistance, and outcome through context-dependent interactions with malignant, stromal, and lymphoid compartments (1–4). Single-cell atlases and CITE-seq in human and murine glioblastoma resolve microglia- versus monocyte-derived TAM programs with conserved lipid-handling, hypoxia-adaptation, and antigen-presentation modules, and reveal stage-specific shifts in lineage composition at recurrence, underscoring the central role of myeloid ecology in progression (5–7). Spatially resolved profiling shows that perivascular, invasive-front, and necrotic niches are enriched for discrete TAM states and immunoregulatory interfaces, linking myeloid topology to effector-cell exclusion and pharmacodynamic heterogeneity (8–10). These data establish myeloid circuits as core—rather than ancillary—determinants of immune failure in glioma.

Mechanistically, glioma–myeloid crosstalk is organized by chemokine/growth-factor axes (recruitment, survival, polarization) and immune checkpoints that constrain phagocytosis and T-cell function. CSF1–CSF1R signaling sustains TAM viability and skews polarization; in proneural glioma models, CSF1R blockade remodels rather than depletes TAMs and attenuates tumor growth, highlighting circuitry that can be pharmacologically reprogrammed (11–13). The CCL2/monocyte-chemoattractant (MCP) family drives CCR2+ monocyte recruitment; when MCPs are perturbed, trafficking is rerouted with CXCL2-dependent neutrophil influx, underscoring chemokine redundancy/plasticity; therefore LR maps should be interpreted as state- and niche-contingent and recomputed under perturbation with compensation-aware statistics (e.g., tracking gain of CXCL2–CXCR2 neutrophil edges when MCPs are blocked) (14–16). Brain-resident microglia maintain CX3CR1-linked tissue residency programs yet acquire disease-associated states in glioma, whereas infiltrating macrophages dominate hypoxic cores and necrotic zones, consistent with spatial specialization of ligand–receptor activity (17–20). Beyond recruitment and maintenance, specific contact and soluble interactions impose immune suppression and invasion: SPP1–CD44 signaling is upregulated in glioblastoma and integrates with STAT3/CEBP-β-driven programs to promote immunoregulatory TAM phenotypes and tumor aggressiveness (21–23); TGF-β signaling further enforces antigen-presentation deficits and exclusionary stromal remodeling; the CD47–SIRPα axis inhibits macrophage phagocytosis; and PD-L1–PD-1 engagement dampens T-cell effector function within TAM-dense niches.

Resolving this crosstalk leverages multi-omics. Single-cell RNA and CITE-seq define sender/receiver cell states; single-cell chromatin profiling adds cis-regulatory logic for myeloid polarization; and spatial proteogenomics/transcriptomics localize active interfaces *in situ* (24–26). Computational frameworks infer and prioritize ligand–receptor (LR) interactions from these data: CellPhoneDB implements a curated, multimeric LR repository with statistical enrichment tests; NicheNet links ligands to downstream gene programs to nominate functional signals; and CellChat models pathway-level communication using a mass-action formulation, enabling comparative analyses across patients, regions, and disease stages (27–29). Comparative benchmarking emphasizes that LR inference should be integrated with spatial context, adjacency-aware metrics (e.g., contact-graph proximity), and chromatin constraints to reduce false positives and to distinguish adjacency-driven signaling from mere co-expression, a requirement that is particularly acute in glioma where microglia and monocyte-derived macrophages co-localize yet differ in ontogeny, accessibility landscapes, and effector coupling (30–32).

In this review, we synthesize the ecology of glioma TAMs and delineate the ligand–receptor architecture that encodes immune evasion across single-cell, chromatin-accessibility, and spatial modalities, with the goal of establishing reproducible analytic standards and translational readouts for myeloid-informed intervention; the purpose of this article is to provide a rigorous, multi-omics framework for mapping TAM–tumor ligand–receptor crosstalk in glioma and specifying how these interactions mechanistically implement immune escape.

## 2 Single-cell and multi-omics map of TAM–tumor crosstalk in glioma

Single-cell atlases resolve TAMs into microglial and monocyte-derived lineages with distinct transcriptional programs and region-specific distributions across glioma. In human tumors, scRNA-seq distinguishes blood-derived macrophages—enriched for immunoregulatory cytokines and altered metabolic pathways—from microglial TAMs that preferentially populate tumor margins, establishing ontogeny as a principal axis of heterogeneity (33–35). Matched scRNA/CITE-seq in patients and mouse models recovers conserved lipid-handling and hypoxia-adaptation modules and delineates dendritic and monocyte/macrophage subsets that vary with disease stage, linking composition to progression (36–39). On the malignant side, glioblastoma cells span dynamic state continua (astrocyte-like, oligodendrocyte-progenitor-like, neural-progenitor-like, mesenchymal-like) that correlate with—and likely respond to—myeloid-derived cues (40, 41). Ligand–receptor inference consistently prioritizes axes that organize this crosstalk: CSF1–CSF1R signaling supports TAM survival and polarization and is pharmacologically reprogrammable in proneural GBM; notably, CSF1R blockade remodels rather than depletes TAMs *in vivo* (42, 43). A second, frequently recovered interface is SPP1 (osteopontin)–CD44, which is enriched at perivascular niches and couples macrophage programs to mesenchymal tumor traits and treatment resistance (44, 45). Integrating these layers with spatially resolved profiling demonstrates that perivascular corridors, invasive fronts, and necrotic cores harbor distinct TAM–tumor exchanges, refining cell-state co-localization into mechanistic, niche-specific signaling maps that explain immune exclusion and heterogeneous drug responses (46–49). **Table 1** shows the principal single-cell and multi-omics modalities, their readouts, the specific questions they address in TAM–tumor communication, and essential controls.

**Table 1:** Core modalities for mapping TAM–tumor ligand–receptor crosstalk in glioma and the inferences each supports.

Modality/analysis step	Typical readout	What it resolves in TAM–tumor crosstalk	Essential controls/caveats
Single-cell RNA-seq (with/without CITE-seq)	Cell states, surface protein abundance (CITE)	Separates microglia- *vs* monocyte-derived TAMs; profiles ligand and receptor expression in TAMs and malignant states	Batch correction across patients; ambient RNA removal; antibody-derived tag background modeling
Tumor-cell state decomposition (e.g., malignant-state scoring)	State probabilities per malignant cell	Links mesenchymal/proneural programs to myeloid-derived cues and niche location	Control for copy-number–driven expression; cross-cohort label transfer validation
Ligand–receptor inference (e.g., CellPhoneDB, NicheNet, CellChat)	Prioritized LR pairs and pathway-level communication	Ranks CSF1–CSF1R, SPP1–CD44, CD47–SIRPα, PD-L1–PD-1, and chemokine axes by sender–receiver specificity	Use curated heteromeric complexes; report database version; compare multiple resources; share code/parameters; hold out spatially co-located nulls
Spatial transcriptomics/proteomics	*In situ* sender–receiver adjacency; pathway activity	Localizes LR activity to perivascular, invasive-front, and necrotic niches; resolves competing myeloid–tumor interfaces	Registration across sections; spot deconvolution uncertainty; concordance with multiplex protein imaging; resolution limits noted
Single-cell ATAC/multiome (RNA+ATAC)	Cis-regulatory accessibility; TF program activity	Tests whether receptors/ligands and downstream programs are chromatin-permitted in TAM and tumor subsets	Peak-to-gene linkage specificity; batch-aware motif enrichment; lineage-matched background
Functional perturbation assays (ex vivo/*in vivo*)	Phagocytosis, cytokine flux, tumor control	Validates causality for ranked LR edges (e.g., CSF1R inhibition, blockade of phagocytosis checkpoints)	Species/model dependence; pharmacodynamic readouts aligned to predicted niche; prioritize *in situ* validation when feasible

Multi-omics integration provides orthogonal evidence for specific immune-evasion circuits. Spatial proteo-genomics that overlays pathway proteins onto transcript-defined states shows that mesenchymal and proneural cores are layered with distinct TAM interfaces and that communication intensity varies across regions, resolving why bulk signatures underperform as biomarkers in GBM (50–52). Spatially organized Notch and hypoxia programs further partition tumor cores and rims, with accompanying shifts in myeloid partners, strengthening the case that LR predictions should be filtered by niche topology to avoid co-expression false positives (11, 53, 54). At the metabolic–innate checkpoint intersection, glioblastoma increases fatty-acid oxidation and upregulates CD47, creating phagocytosis-proof tumor cells; phagocytic checkpoints on TAMs can be overcome only when innate signals are combined with genotoxic or STING agonism, aligning LR maps with actionable dependencies (55, 56). Systematic benchmarking of communication tools indicates that database coverage and model assumptions materially change predicted edges—including sensitivity to database versioning, receptor isoforms, and spatial resolution—recommending consensus across complementary resources, parameter sharing, and pathway-aware statistics when prioritizing LR pairs for perturbation.

These single-cell, chromatin, and spatial layers converge on a reproducible architecture in which ontogeny-defined TAM programs and state-defined tumor programs engage through a limited set of crosstalk axes—CSF1/CSF1R for maintenance and polarization, SPP1–CD44 for mesenchymal reinforcement and perivascular conditioning, and CD47–SIRPα and PD-L1–PD-1 as dominant effector brakes—whose intensity and topology vary by niche.

## 3 Spatial ecosystems and circuit topology of immune evasion in glioma

**Figure 1:**
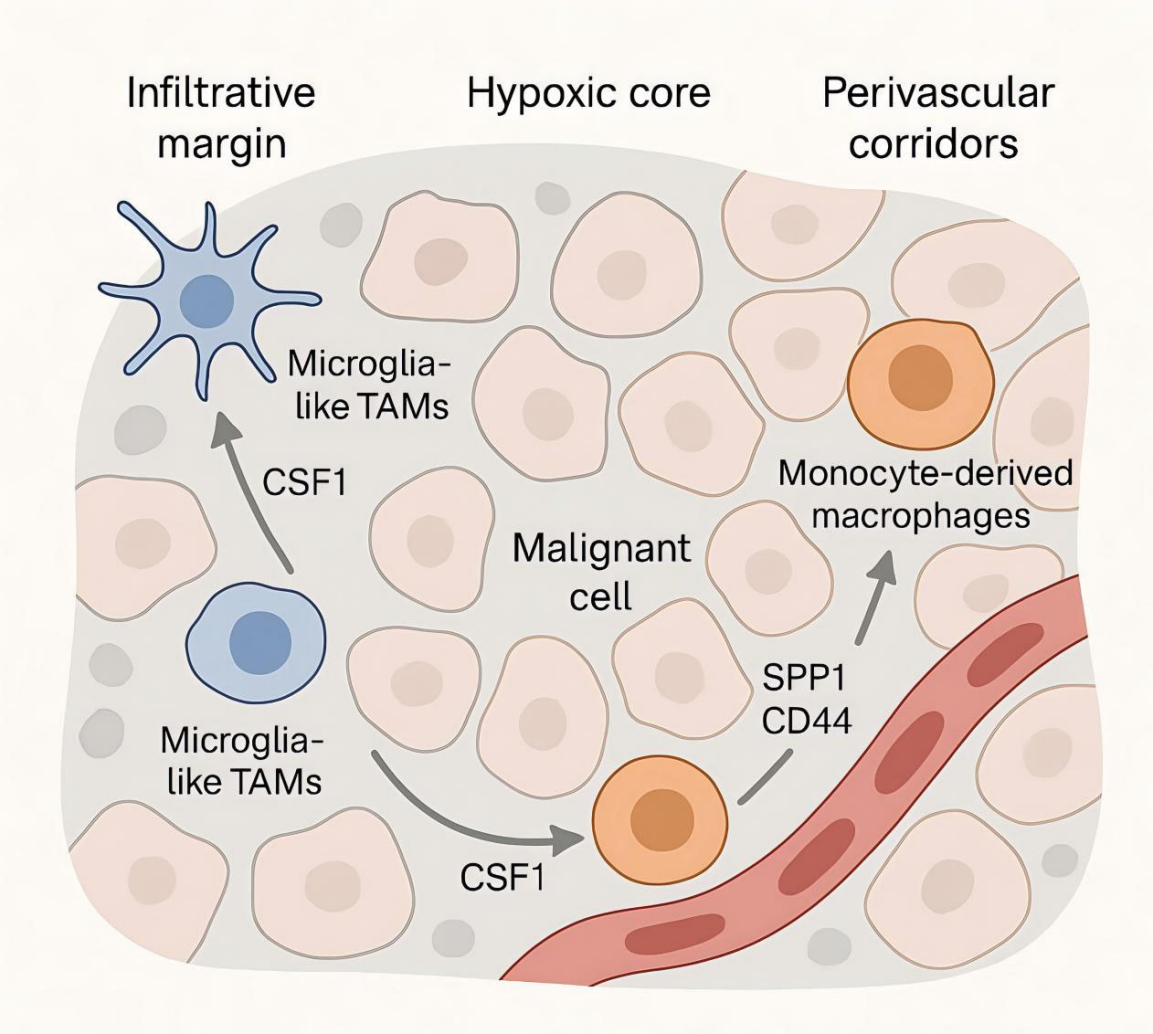
Spatial niches and signaling of tumor-associated macrophages in the glioma microenvironment—margins vs cores.

Spatially resolved maps in glioma demonstrate that immune evasion is encoded by niche-specific adjacency between ontogeny-defined TAM states and malignant programs. As shown in **Figure 1**, microglia-like TAMs preferentially accumulate at infiltrative margins, whereas monocyte-derived macrophages dominate hypoxic cores; multiplex imaging and spatial transcriptomics further resolve immune neighborhoods associated with outcome in glioblastoma, including survival-linked myeloperoxidase-positive macrophage subsets and variable lymphoid access at tumor borders (57–59). Perinecrotic regions show the strongest immunosuppressive signatures with dense myeloid content, while perivascular corridors exhibit distinct inflammatory and angiogenic signaling, indicating that spatial topology—not bulk abundance—governs checkpoint engagement and effector exclusion.

These ecosystems are organized by a limited set of ligand–receptor circuits whose activity depends on location and lineage. Single-cell atlases across species and disease stages define competition and specialization between microglia- and monocyte-derived TAMs, providing the sender–receiver context for niche-restricted signaling (60, 61). Within perivascular corridors, infiltrating monocyte-derived and border-associated/perivascular macrophages are the dominant SPP1 producers (microglia contribute less); SPP1—both secreted and matrix-bound (ECM-tethered)—engages CD44 on mesenchymal-shifted glioma cells and endothelial/pericyte compartments; CD44 isoform usage (CD44s with context-dependent CD44v6/v10) and integrin co-receptors (e.g., αvβ3/αvβ5) tune adhesion/angiogenic outputs that reinforce mesenchymal programs (62, 63). Hypoxic and mesenchymal zones are enriched for HMOX1^+^ myeloid cells that release IL-10 and drive spatially localized T-cell dysfunction through JAK/STAT-dependent programs, providing a cytokine circuit that couples TAM proximity to effector exhaustion (64, 65). A complementary metabolic checkpoint is topologically organized by CD39^+^ microglia adjacent to CD73^+^ tumor cells, producing adenosine-rich interfaces that suppress antitumor immunity; the strength of CD39–CD73 co-localization correlates with adverse clinical features, underscoring a spatially constrained purinergic pathway of immune escape.

These data support a circuit topology in which perivascular SPP1–CD44 and angiogenic signals consolidate mesenchymal programs and vascular remodeling; perinecrotic hubs concentrate IL-10–dominated myeloid signaling and adenosine metabolism; and border zones variably permit lymphoid ingress depending on TAM continuity at tumor–stroma interfaces (55, 66, 67). This model explains why co-expression overestimates communication: signaling requires chromatin-permitted receptors in adjacent receiver states within specific niches. It also nominates quantitative spatial readouts—macrophage–tumor interface length, CD39–CD73 proximity, and enrichment of IL-10–linked HMOX1^+^ myeloid neighborhoods—as mechanistic biomarkers to benchmark interventions that reprogram TAMs, disrupt perivascular SPP1–CD44, or attenuate purinergic and cytokine checkpoints in glioma.

## 4 Translational readouts and interventions: biomarkers and myeloid-directed therapeutics

Translational readouts should quantify myeloid–tumor LR activity and spatial deployment. Tissue biomarkers that index SPP1^+^ TAM programs and perivascular SPP1–CD44 signaling associate with mesenchymal traits and poor outcome in glioma and can be captured by RNA panels or multiplexed protein assays (40, 68, 69); spatial adjacency metrics such as CD39^+^ myeloid–CD73^+^ tumor proximity and enrichment of HMOX1^+^ IL-10–secreting myeloid neighborhoods provide orthogonal evidence of immunosuppressive niches and should be prospectively standardized as pharmacodynamic endpoints (39, 70, 71). Circulating readouts that reflect myeloid trafficking complement tissue metrics; in glioma models, IL-8 neutralization enhances the efficacy of immune checkpoint blockade, supporting baseline and on-treatment IL-8 as a negative biomarker and as a targetable axis (16, 19, 72). A composite panel that integrates SPP1–TAM burden, CD39–CD73 spatial proximity, HMOX1/IL-10 myeloid niches, and IL-8 levels can report on the intensity and topology of myeloid circuits that gate effector function and should be embedded in trial schemas evaluating myeloid-directed agents.

Therapeutic strategies that directly modulate these circuits are feasible and should be layered onto contemporary glioma backbones with predefined mechanistic endpoints. CSF1R blockade remodels, rather than depletes, TAMs in proneural glioma models and constrains tumor growth, establishing a paradigm of pharmacologic reprogramming of maintenance signals; subsequent studies corroborate antitumor effects of CSF1R inhibition and highlight context dependence that argues for biomarker-guided selection (15, 73). Myeloid checkpoint inhibition at the phagocytosis axis is mechanistically justified: the CD47–SIRPα pathway suppresses macrophage effector function across solid tumors, yet in glioma, CD47 blockade shows limited activity as monotherapy and demonstrates improved phagocytic and antitumor effects when combined with genotoxic stressors, supporting rational combinations with standard therapy or opsonizing antibodies. Targeting macrophage-intrinsic signaling can convert suppressive programs: PI3Kγ functions as a switch enforcing immunosuppressive transcriptional states in myeloid cells; its inhibition restores inflammatory outputs and synergizes with PD-1 blockade in preclinical models, nominating PI3Kγ inhibitors for evaluation in glioma with embedded myeloid pharmacodynamics (57, 74, 75). Purinergic signaling is a spatially organized checkpoint: co-localization of CD73^+^ tumor cells with CD39^+^ microglia amplifies extracellular adenosine that suppresses T cells via high-affinity A2A receptors and conditions myeloid cells via lower-affinity A2B; hypoxia/HIF-1α upregulates CD73, strengthening this axis—rationalizing anti-CD73 or adenosine-receptor antagonists with spatial biomarkers as inclusion/response criteria. Trial designs should incorporate on-treatment reduction of SPP1–TAM signatures, attenuation of CD39–CD73 proximity, depletion or reprogramming of HMOX1^+^ IL-10 niches, and restoration of effector cell access as primary pharmacodynamic endpoints to attribute benefit to myeloid modulation.

## 5 Outlook and standards for integrative ligand–receptor multi-omics in glioma

Integrative ligand–receptor (LR) profiling in glioma should progress from descriptive atlases to standardized, decision-oriented pipelines that co-register single-cell RNA (± CITE-seq), single-cell chromatin accessibility (scATAC or multiome), and spatial readouts with harmonized metadata, tissue-region annotation, and predefined endpoints (11, 76, 77). A practical state dictionary for sender/receiver modeling is justified by existing atlases and should minimally include microglia-derived and monocyte-derived TAM programs, mesenchymal/proneural malignant states, and region-specific endothelial and stromal compartments (78–81); these choices are supported by foundational glioblastoma single-cell studies resolving malignant state continua and myeloid ontogeny, and by datasets that map myeloid subset diversity and spatial bias in experimental and human gliomas.

In silico LR inference should use curated, heteromer-aware resources—databases that encode multi-subunit stoichiometry (e.g., α/β chains) and isoform-specific binding (e.g., CD44 variants)—with transparent statistics, and should favor consensus across complementary tools. CellPhoneDB tests enrichment of multimeric complexes (enrichment paradigm), NicheNet scores ligands by regulatory potential on target programs (regulatory-potential paradigm), and CellChat estimates pathway-level mass-action flow (mass-action paradigm); when outputs disagree, treat two-of-three concordance (plus spatial/chromatin support) as high confidence and reserve tool-unique calls for exploratory validation. Cross-tool agreement should be explicitly reported, and permutation-based nulls should reflect tissue structure (e.g., region-stratified or spatially permuted cells) rather than only global label shuffling (4, 82, 83). Edges should be ‘gated’ by scATAC/multiome evidence: (i) receiver-side promoter/enhancer accessibility with peak-to-gene linkage for the receptor (e.g., co-accessibility/correlation above a preset threshold), (ii) activity of downstream TF motifs (e.g., chromVAR/GSVA deviation > ~1–2 SD), and (iii) sender-side accessibility for the ligand; applying these filters materially reduces co-expression false positives, with accepted edges summarized at the donor–receiver–pathway level.

Spatial registration is required to distinguish adjacency-dependent signaling from mere co-abundance. Spatial transcriptomics and multiplexed imaging should localize LR activity to perivascular, invasive-front, and perinecrotic ecosystems and quantify: (a) LR intensity—unitless, per donor–receiver pair mass-action–style scores (normalized ligand × receptor per cell); (b) communication burden—we define this as per-niche or patient-level aggregates (sum/mean of accepted edges normalized by sender/receiver counts); and (c) proximity scores—adjacency-aware metrics (interface length, inverse distance or co-localization indices on a contact graph) computed within regions (55, 61, 84). In glioma, perivascular SPP1–CD44 signaling and mesenchymal programs, and the purinergic CD39–CD73 interface between microglia and tumor cells, are recurrently enriched in defined niches; spatial frameworks that quantify these arrangements have been associated with prognostic and biological stratification and therefore constitute suitable benchmarks for LR pipelines.

Method reporting should include pre-analytical variables (fixation, dissociation, steroid exposure), an explicit region map (core, rim, invasive margin, perivascular, perinecrotic), doublet and ambient-RNA handling, batch correction strategy, and cross-cohort label transfer performance. Minimal LR-specific reporting should enumerate database versions, multimer handling, null model specification, per-edge effect sizes and false-discovery rates, and the criteria for chromatin gating. Spatial sections should include segmentation and registration procedures, spot deconvolution uncertainty, and cross-modality concordance (85–87). Where possible, open-source code and parameter files should be deposited with derived matrices so that LR calls can be recomputed without raw data access.

Functional validation should be embedded early using positive-control axes that are established in glioma and that exercise distinct mechanistic classes. CSF1–CSF1R can benchmark maintenance/polarization signals; SPP1–CD44 can benchmark perivascular mesenchymal reinforcement; and CD39–CD73 can benchmark metabolic checkpoint topology (56, 88, 89). Small-scale perturbations (ex vivo receptor blockade, phagocytosis/cytokine readouts) should be aligned to sender/receiver states predicted by the pipeline and, for spatial claims, should preferentially sample regions where adjacency scores are highest.

Clinical translation should move from single markers to composite communication scores with spatial context. A pragmatic panel for early-phase studies would combine: (i) a communication burden for perivascular SPP1–CD44 and a mesenchymal-state readout in tumor cells; (ii) a spatial purinergic-axis metric quantifying CD39^+^ microglia–CD73^+^ tumor proximity; and (iii) circulating or tissue chemokine indices that track myeloid trafficking. Practical hurdles include tissue accessibility for region-resolved assays, assay standardization across platforms, and turnaround times compatible with clinical decision-making. Prospective designs should prespecify pharmacodynamic success as reduction of targeted communication scores with concomitant restoration of effector access, and should stratify by IDH status and steroid exposure to minimize confounding.

Benchmarking should use multi-institutional samples with repeated sections from the same tumor to measure technical and biological variance, and should include orthogonal perivascular-interactome or spatial-omics datasets as “hold-out” validation. Published glioma spatial studies already provide suitable templates for region-matched validation and for evaluating whether LR calls generalize across platforms and centers; future releases should prioritize needle-biopsy–compatible protocols to facilitate clinical implementation.
